# Incidence of acute kidney injury during pregnancy and its prognostic value for adverse clinical outcomes: A systematic review and meta-analysis

**DOI:** 10.1097/MD.0000000000029563

**Published:** 2022-07-29

**Authors:** Thananda Trakarnvanich, Tanun Ngamvichchukorn, Paweena Susantitaphong

**Affiliations:** a Division of Nephrology, Department of Medicine, Faculty of Medicine, Vajira Hospital, Navamindradhiraj University, Bangkok, Thailand; b Division of Nephrology, Department of Medicine, Faculty of Medicine, King Chulalongkorn Memorial Hospital, Bangkok, Thailand; c Research Unit for Metabolic Bone Disease in CKD patients, Faculty of Medicine, Chulalongkorn University, Bangkok, Thailand.

## Abstract

**Background:**

Acute kidney injury (AKI) that develops during pregnancy results from pregnancy-induced hypertension, hemorrhage, and sepsis, associated with morbidity and mortality in the fetus and mother. This meta-analysis was conducted to evaluate the incidence of pregnancy-related AKI (PR-AKI) and adverse clinical outcomes.

**Methods:**

PubMed and Scopus were systematically searched for studies published between 1980 and 2021. We included cross-sectional, retrospective, and prospective cohort studies that reported the incidence of PR-AKI as well as adverse fetal and maternal clinical outcomes. A random-effects model meta-analysis was performed to generate summary estimates.

**Results:**

The meta-analysis included 31 studies (57,529,841 participants). The pooled incidence of PR-AKI was 2.0% (95% confidence interval [CI] 1.0–3.7). Only 49.3% of patients received antenatal care. The most common cause of PR-AKI was preeclampsia (36.6%, 95% CI 29.1–44.7). The proportion of patients requiring hemodialysis was 37.2% (95% CI 26.0–49.9). More than 70% of patients had complete recovery of renal function, while 8.5% (95% CI 4.7–14.8) remained dependent on dialysis. The pooled mortality rate of PR-AKI was 12.7% (95% CI 9.0–17.7). In addition, fetal outcomes were favorable, with an alive birth rate of 70.0% (95% CI 61.2–77.4). However, the rate of abortion and/or stillbirth was approximately 25.4% (95% CI 18.1–34.4), and the rate of intrauterine death was 18.6% (95% CI 12.8–26.2).

**Conclusions:**

Although the incidence of PR-AKI is not high, this condition has a high impact on morbidity and mortality in both fetal and maternal outcomes. Early prevention and treatment from health care professionals are needed in PR-AKI, especially in the form of antenatal care and preeclampsia medication.

## 1. Introduction

Acute kidney injury (AKI) that develops during pregnancy results from such as pregnancy-induced hypertension, hemorrhage, and sepsis, associated with morbidity and mortality in the fetus and mother.^[1,2]^ Previous studies have reported a dramatic reduction in the incidence of AKI during pregnancy in developed countries, from 1/3000 to 1/20,000, which was attributed to improved antenatal care.^[3]^ The diagnosis and treatment of AKI during pregnancy (pregnancy-related AKI, PR-AKI) is challenging. The risk profiles and geographic distribution of pregnancy-related AKI may be different in various parts of the world^[4–6]^ and between different regions within the same country. The reported incidence of PR-AKI in developing countries, such as India and Pakistan, is 0.02% to 71.5%.^[7–10]^ Data from South Asia suggest that PR-AKI accounts for 10% of total AKI cases.^[5]^ However, in high-income countries, PR-AKI is rare.^[11]^

In developing countries, sepsis and hemorrhage account for >50% of cases of PR-AKI,^[12,13]^ in contrast to developed countries, where chronic hypertension, renal disease, and preeclampsia are important causes of this condition.^[14,15]^ The maternal and fetal outcomes that are of concern include permanent damage to the kidney, maternal survival, rate of renal recovery, dialysis dependence, rate of live births, stillbirths, and intrauterine death. This systematic review and meta-analysis of observation studies aimed to evaluate the overall incidence of PR-AKI and its prognostic value for fetal and maternal adverse clinical outcomes.

## 2. Methods

### 2.1. Search strategy and study selection

We performed a literature search in MEDLINE and SCOPUS (from 1989 to March 2021) to identify eligible studies using the Medical Subject Heading and text keywords “acute renal failure”, “acute kidney injury”, “acute renal insufficiency”, combined with all spellings of “pregnancy” and “pregnant.” The search was limited to the English language. Reference lists of the obtained articles were also searched for relevant publications. Only officially published papers were considered for inclusion in the present meta-analysis. Studies were required to meet the following inclusion criteria: original article including cross-sectional, retrospective, or prospective cohort studies, and reported pregnancy outcomes and kidney outcomes in women with PR-AKI. Narrative reviews, case reports/series, conference abstracts, and registry databases were excluded. We followed the preferred reporting items for systematic review and meta-analysis and meta-analyses Of observational studies in epidemiology guidelines to report the meta-analysis (Table 1, Supplemental Digital Content, http://links.lww.com/MD/G956).^[16]^

### 2.2. Data extraction

Data were independently extracted from full-text articles by 2 investigators (T.T. and T.G.) using a standardized approach. Disagreement between the 2 investigators was recorded and adjudicated by a third reviewer (P.S.). Data on country of origin, number of patients, patient age, number of parities, trimester, causes of AKI, and underlying diseases were recorded. We also extracted data on maternal and fetal outcomes, number of patients who required renal replacement therapy, recovery of renal function, and subsequent dependence on dialysis. The other maternal outcomes included death; preeclampsia; hemorrhage during and after delivery; and hemolysis, elevated liver enzymes, and low platelet count (HELLP) syndrome. Fetal outcomes, including perinatal death, abortion, stillbirth, and preterm delivery, were also recorded.

### 2.3. Study quality assessment

The quality of the cohort studies was evaluated by using the Newcastle-Ottawa Scale, which allocates a maximum of 9 points for quality of the selection, comparability, and outcome of study populations. Study quality scores were defined arbitrarily as poor (0–3), fair (4–6), or good (7–9).

### 2.4. Statistical analysis

Random-effects model meta-analyses were conducted to generate pooled incidence rates of AKI, caused of AKI, fetal, and maternal outcomes. All pooled estimates are provided with 95% confidence intervals (95% CIs). Heterogeneity was assessed using the *I*^2^ index. The *I*^2^ index describes the percentage of total variation across studies due to true heterogeneity rather than chance, with a value of >75% indicating medium-to-high heterogeneity. To examine the effect of developed vs under developed countries on the incidence of AKI, we conducted random-effects subgroup. We also performed subgroup analysis on the AKI definitions by using the RIFLE or Kidney Disease Improving Global Outcomes (KDIGO) vs other definitions including biochemical/urine output/dialysis requirement-based definitions/administrative codes for AKI derived from the International Classification of Diseases, 9th or 10th Revision, Clinical Modification methodology. We explored the robustness of interested outcomes by sensitivity analysis and subgroup analysis based on study quality. Publication bias was formally assessed using the Egger test. We performed the meta-analyses using Comprehensive Meta-Analysis (version 2.0; Biostat, www.meta-analysis.com).

## 3. Results

**Figure 1.**
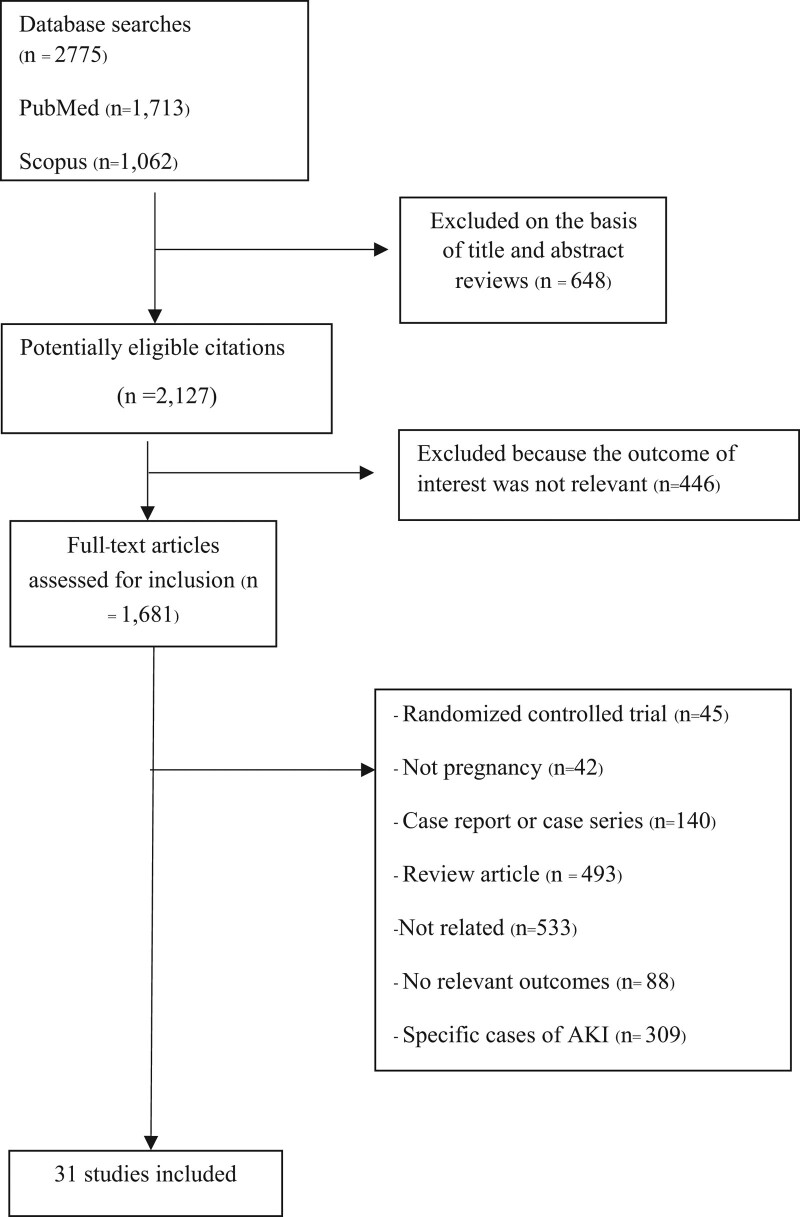
Flow diagram of articles considered for inclusion.

The literature search yielded 2775 articles; 1681 studies were retrieved for detailed evaluation, among which 31 fulfilled the inclusion criteria (Fig. 1). Table 1 summarizes the characteristics of the included studies. These studies were performed between 1993 and 2021, with sample sizes ranging from 75 to 42,190,790 participants. The scope of the study covered all regions of the world. Most studies were conducted in India (n = 10), followed by the United States (n = 4); China and Pakistan (n = 3 each); Brazil, Morocco, and Canada (n = 2 each); and Mexico, Turkey, Egypt, Greece, and Malawi (n = 1 each; Table 1).

**Table 1:** Characteristics of the included studies in the meta-analysis.

	**Authors**	**Publication years**	**Study design**	**Country**	**No. of AKI**	**No. of patients**	**Mean age**	**AKI criteria**	**Study quality**
1	Alexopoulos et al^[24]^	1993	Retrospective study	Greece	18	200	32.0	Serum creatinine >1.8 mg/dL	6.0
2	Naqvi et al^[38]^	1996	Cross-sectional	Pakistan	43	238	28.30	Not stated	6.0
3	Selcuk et al^[39]^	1998	Retrospective cohort	Turkey	74	487	29.0	Not stated	6.0
4	Najar et al^[3]^	2008	Prospective cohort	India	40	569	28.94	Urine output <400 mL or creatinine >1.5 mg/dL	7.0
5	Ansari et al^[40]^	2008	Retrospective study	Pakistan	42	116	29.25	Not stated	6.0
6	Goplani et al^[22]^	2008	Prospective cohort	India	70	772	25.60	Urine output <400 mL or creatinine >2.0 mg/dL	8.0
7	Silva Jr et al^[2]^	2009	Cross-sectional	Brazil	55	63,882	26.2	RIFLE	9.0
8	Prakash et al^[5]^	2010	Prospective cohort	India	85	4758	27.15 ± 4.66	Not stated	7.0
9	Chaudhri et al^[41]^	2011	Retrospective	Pakistan	51	346	28.0	Not stated	6.0
10	Bentata et al^[42]^	2012	Retrospective cohort	Morocco	46	22,320	29.50 ± 6.0	RIFLE	9.0
11	Gurrieri et al^[43]^	2012	Retrospective study	United States	54	13,372	28.0	AKIN	7.0
12	Arrayhani et al^[44]^	2013	Prospective observational	Morocco	37	5600	29.03	RIFLE	7.0
13	Godara et al^[37]^	2014	Prospective cohort	India	57	580	26.4	Not stated	8.0
14	Mehrabadi et al^[45]^	2014	Retrospective cohort	Canada	502	2,193,425	Not stated	Not stated	6.0
15	Gopalakrishnanet al^[15]^	2015	Prospective observational	India	130	1668	25.4 ± 4.73	Urine output <400 mL or creatinine increased 1.5 times from the baseline	8.0
16	Hildebrand et al^[4]^	2015	Retrospective cohort	Canada	188	1,918,789	30.0	Not stated	8.0
17	Liu et al^[34]^	2015	Retrospective cohort	China	22	18,589	30.9 ± 5.4	KDIGO	9.0
18	Vineet et al^[46]^	2016	Cross-sectional	India	52	570	26.2	Urine output <400 mL or creatinine >2.0 mg/dL	6.0
19	Mehrabadi et al^[45]^	2016	Retrospective cohort	United States	4300	10,969,263	29.90	Not stated	9.0
20	Prakash et al^[14]^	2016	Retrospective observational	India	259	3100	29.80	Serum creatinine >1.0 mg/dL, oligoanuria >12 h, need for dialysis	7.0
21	Ibarra-Hernandez et al^[47]^	2017	Cross-sectional	Mexico	18	75	24.80	RIFLE	8.0
22	Huang et al^[30]^	2017	Retrospective study	China	343	42,173	29.41 ± 5.94	Serum creatinine >70.72 µmol/L	8.0
23	Silva Junior^[48]^	2017	Cross-sectional	Brazil	92	389	27.10	Not stated	7.0
24	Tangren et al^[10]^	2017	Prospective observational	United States	246	46,646	31.0 ± 6.3	KDIGO	9.0
25	Mir et al^[52]^	2017	Retrospective	India	61	713	26.10 ± 4.3	KDIGO	7.0
26	Mahesh et al^[20]^	2017	Prospective observational	India	165	10,576	25.0	RIFLE	7.0
27	Cooke et al^[49]^	2018	Prospective observational	Malawi	26	332	26.50	KDIGO	9.0
28	Prakash et al^[9]^	2018	Prospective cohort	India	132	4741	26.80	Serum creatinine >1.0 mg/dL, oligoanuria >12 h, need for dialysis	7.0
29	Liu et al^[50]^	2019	Retrospective cohort	China	795	10,920	Not stated	KDIGO	9.0
30	Shah et al^[53]^	2020	Retrospective cohort	United States	32,385	42,190,790	28.0	Not stated	7.0
31	Gaber et al^[54]^	2021	Prospective observational	Egypt	40	4000	28.7 ± 5.9	KDIGO	6.0

The etiology of PR-AKI was multifactorial in many patients. Almost all studies were retrospective cohort studies and were from a single center. The definition of PR-AKI varied considerably across these studies—1 study defined PR-AKI as increase in serum creatinine (Scr) level by approximately 1.5 times from baseline or decrease in urine output to<400 mL; 6 studies defined AKI based on the KDIGO criteria; 5 studies defined AKI according to the RIFLE classification, 1 study defined AKI based on the Acute Kidney Injury Network classification; 7 studies defined PR-AKI according to the level of creatinine, 3 studies defined PR-AKI as Scr level>1.0 mg/dL, 1 study defined PR-AKI as Scr level >1.5 mg/dL, 1 study defined PR-AKI as Scr level >1.8 mg/dL, and 2 studies defined PR-AKI as Scr level >2.0 mg/dL. The remaining 11studies did not specify criteria for diagnosing AKI.

### 3.1. Characteristics of the studies

In the meta-analysis of 31 studies, the pooled mean age of the participants was 28.2 years (95% CI 27.6–28.8; *I*^2^ = 98.5%). By meta-analysis, 36.1% (95% CI 22.9–51.8, 18 studies, *I^2^* = 98.4%) of patients were primigravida, while 56.9% (95% CI 52.0–61.7, 17 studies, *I^2^* = 78.0%) of patients were multigravida. In term of onset of AKI, 12.6% (95% CI 7.3–20.8, 11 studies, *I^2^* = 86.3%), 15.7% (95% CI 9.7–24.2, 11 studies, *I^2^* = 84.5%), 69.0% (95% CI 51.5–82.3, 13 studies, *I^2^* = 96.1%), and 48.6% (95% CI 36.3–61.2, 8 studies, *I*^2^ = 86.0%) of patients presented with PR-AKI during the first trimester, second trimester, third trimester, and postpartum period, respectively. Only 49.3% of patients received antenatal care (95% CI 25.9–72.9, 6 studies, *I^2^* = 94.9%).

### 3.2. Incidence and causes of PR-AKI during pregnancy

**Figure 2.**
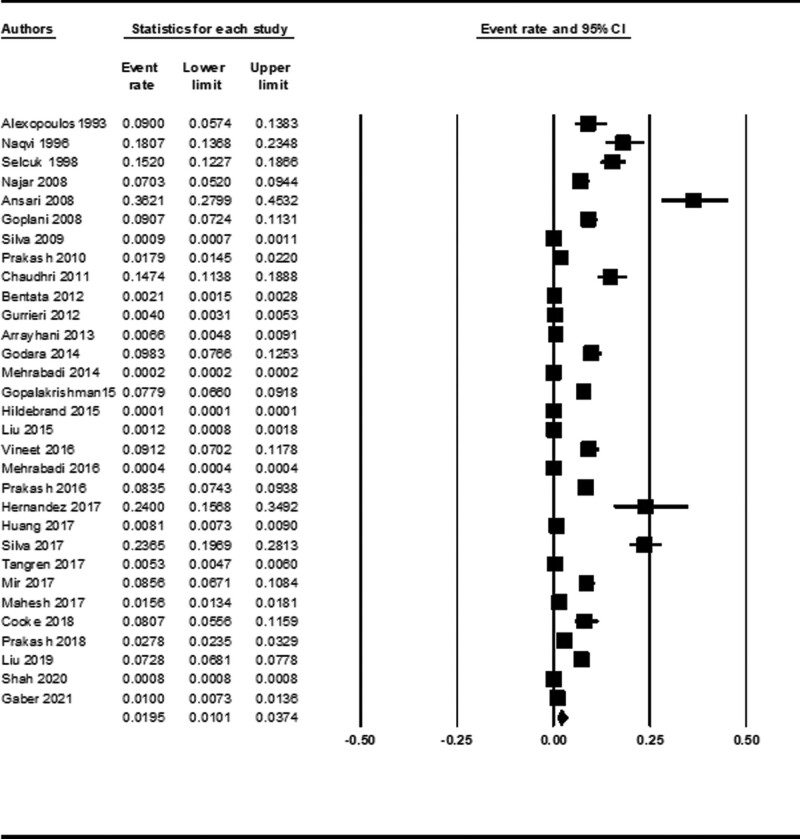
Forest plot of incidence of PR-AKI. CI = confidence interval, PR-AKI = pregnancy-related acute kidney injury.

The incidence of AKI during pregnancy and puerperium was 1.95% (95% CI 1.01–3.73) using data from 31 studies (Fig. 2). By meta-analysis of 29 studies, 36.6% (95% CI 29.1–44.7, *I^2^* = 96.8%) patients had preeclampsia and hypertensive disorders during pregnancy, 21.0% (95% CI 12.8–32.5, 18 studies, *I^2^* = 96.5%) had septic abortion, 16.6% (95% CI 9.1–28.4, 24 studies, *I^2^* = 98.4%) had other causes or unknown causes of abortion, 15.1% (95% CI 11.5–19.6, 24 studies, *I^2^* = 88.1%) had antepartum hemorrhage, 14.1% (95% CI 5.6–31.4, 3 studies, *I^2^* = 74.7%) had acute gastroenteritis, 13.3% (95% CI 9.3–18.7, 13 studies, *I^2^* = 80.4%) had HELLP syndrome, 13.2% (95% CI 9.7–17.8, 28 studies, *I^2^* = 91.3%) had postpartum hemorrhage, 5.8% (95% CI 3.6–9.1, 29 studies, *I^2^* = 92.1%) had acute pyelonephritis, 4.5% (95% CI 2.6–7.5, 6 studies, *I^2^* = 59.1%) had acute fatty liver of pregnancy, and 3.5% (95% CI 1.3–8.7, 6 studies, *I^2^* = 82.5%) had glomerular disease.

### 3.3. Maternal outcomes

In the meta-analysis of 25 studies, the pooled mortality rate was an average of 12.7% (Table 2). Ten studies reported the cause of maternal death, including sepsis, antepartum hemorrhage, preeclampsia/HELLP syndrome, and adult respiratory distress syndrome.

**Table 2:** Summary of maternal and fetal outcomes.

**Outcomes**	**No. of studies**	**Event rate% (95% CI**)	***I* ^2^ index (%**)	**Egger test *P* value**
Maternal outcomes				
Complete recovery	25	70.6% (62.7–77.5)	89.6	.52
Partial recovery	22	14.7% (10.1–21.0)	86.0	.01
Dialysis dependence	15	8.5% (4.7–14.8)	84.0	.050
Required hemodialysis	27	37.2% (26.0–49.9)	98.7	.001
Required peritoneal dialysis	7	10.0% (5.9–16.5)	75.3	.07
Required both modalities	4	8.2% (4.1–15.7)	53.0	.12
Mortality	25	12.7% (9.0–17.7)	96.3	.001
Fetal outcomes				
Live births	21	70.0% (61.2–77.4)	93.4	.80
Intrauterine death	11	18.6% (12.8–26.2)	76.5	.12
Preterm	4	28.5% (14.7–48.1)	90.5	.05
Perinatal mortality	20	25.4% (18.1–34.4)	94.6	.78

Furthermore, 70.6% of patients with PR-AKI achieved complete recovery,14.7% achieved partial recovery, and 8.5% remained dependent on dialysis. In terms of dialysis modality in patients with PR-AKI, 37.2% of patients required hemodialysis (HD), 10.0% required peritoneal dialysis (PD), and 8.2% required both HD and PD due to inadequate ultrafiltration by PD alone. The indications for HD included oligo/anuria, hyperkalemia, uremia, metabolic acidosis, and acute pulmonary edema.

### 3.4. Fetal outcomes

By the meta-analysis, 70.0% of patients had live births (Table 2). The incidence of intrauterine death was 18.6%. The offspring of mothers with PR-AKI were born earlier, with a pooled rate of 28.5%. The mortality rate among perinatal infants was 25.4%.

### 3.5. Investigation of heterogeneity

In subgroup analysis according to developed vs underdeveloped countries (definition by World Economic Situation and Prospects) on the incidence of AKI, the pooled incidence of PR-AKI in developed countries was 0.14% (95% CI 0.08%–0.23%; Table 3). Surprisingly, the high pooled incidence of PR-AKI was observed in underdeveloped countries (4.11%; 95% CI 2.38%–7.01%). In terms of AKI definition, based on RIFLE or KDIGO criteria, the pooled incidence of PR-AKI was 1.31% (95% CI 0.47%–3.61%), while the high pooled incidence of PR-AKI was reported in studies that used other definitions (2.42%; 95% CI 1.21%–4.79%).

**Table 3:** Subgroup analysis of incidence of PR-AKI.

**Outcomes**	**No. of studies**	**Event rate (95% CI**)	***I*^2^ index**
Incidence of PR-AKI	31	1.95% (1.01–3.73)	99.94
Subgroup analysis based on			
Country classification*			
Developed economies	7	0.14% (0.08–0.23)	99.87
Developing economies	24	4.11% (2.38–7.01)	99.48
AKI definition			
RIFLE/KDIGO	11	1.31% (0.47–3.61)	99.67
Others	20	2.42% (1.21–4.79)	99.94
Study quality			
Poor and fair	8	5.59% (0.35–49.71)	99.92
Good	23	1.35% (0.49–3.67)	99.94

We performed a sensitivity analysis to assess the consistency of the results based on the study quality; the pooled incidence of PR-AKI was 1.35% (95% CI 0.49–3.68) after excluding the study that had poor and fair quality. The high incidence of PR-AKI was observed in poor and fair study quality.

### 3.6. Publication bias

As the Egger test results, most of the interested outcomes were mainly insignificant (Table 2).

## 4. Discussion

In this meta-analysis of >58 million pregnancies, the incidence of AKI during pregnancy was 2%. AKI during pregnancy is associated with increased morbidity in the mother and fetus. The incidence of AKI varies based on the geographical area, with most studies conducted in the Indian subcontinent. Since the time span of study inclusion was >30 years, various definitions of AKI were used. Recently, the KDIGO criteria have been widely adopted by professional societies as the standardized criteria for AKI. The criteria provide a much higher estimated incidence (10-fold increase) of AKI than Code-Classification AKI.^[17,18]^

The physiological changes and increase in glomerular filtration rate with the reduction in Scr levels during pregnancy are associated with increased difficulty for early and accurate diagnosis of PR-AKI.^[19]^ In previous studies, the incidence of PR-AKI was 7% to 9 % in the Indian subcontinent,^[20]^ 2.5% in China,^[21]^ and 1% to 28% in developed countries. Our meta-analysis demonstrated that the pooled incidence of PR-AKI in developed countries was only 0.14%. Unfortunately, the high pooled incidence of PR-AKI (4.11%) was observed in underdeveloped countries. Only 49.3% of patients received antenatal care. Therefore, antenatal care should be emphasized in public health care to prevent septic abortion and pregnancy-induced hypertension, especially in underdeveloped countries.

In this study, approximately 50% of patients developed postpartum PR-AKI. Similarly, Sivakumar et al^[22]^ reported that 74.5% of patients developed PR-AKI during the postpartum period. However, most of the studies showed predominant incidence of PR-AKI in the trimester. In our meta-analysis, the pooled AKI during the first trimester, second trimester, third trimester, and postpartum were reported at 12.6%, 15.7%, 69%, and 48.6%, respectively. Improvements in antenatal care and early referral to nephrologists to decrease the risk of kidney injury in PR-AKI should be highlighted. The mean age of our patients was 28.2 years.^[22–26]^ In a study by Kabbali et al,^[27]^ age >38 years was significantly associated with unfavorable outcomes and increased perinatal complications. Therefore, the pooled incidence of PR-AKI in this meta-analysis might be underestimated, especially in advanced maternal age.

Preeclampsia and pregnancy-related hypertension were responsible for 36.6% of PR-AKI cases in our study. Preeclampsia is one of the most important causes of AKI during pregnancy, especially in Indian studies^[11]^; however, most patients with this condition do not develop AKI.^[28]^ The frequency of AKI in preeclampsia varied between 24% and 35% in different centers in India.^[11,29]^ This was the most frequent cause of PR-AKI in this meta-analysis. Preeclampsia is also the main cause of severe PR-AKI and typically occurs during the third trimester and postpartum period.^[30]^ The frequency of preeclampsia ranges between 2% and 7% in healthy nulliparous women and increases substantially in women with multiple gestations, chronic hypertension, diabetes mellitus, and chronic kidney disease.^[31]^ The effect of preeclampsia on endothelial injury and vasoconstriction, which would be expected to lower the glomerular filtration rate,^[32,33]^ may affect long-term renal function. The causes of PR-AKI have changed over time from septic abortion in the past to preeclampsia and hemorrhagic shock in the present era due to increased attention of health care professionals to prenatal cases.

The HELLP syndrome is a serious disorder in pregnancy, accounting for 15% to 40% of all cases of PR-AKI^[34]^ and up to 54% to 60% of severe cases.^[35]^ The HELLP syndrome is considered a continuum of preeclampsia. In our study, HELLP syndrome accounted for 13.3% of total PR-AKI cases, which was a similar rate associated with other causes, such as acute gastroenteritis, septic abortion, postpartum hemorrhage, and acute pyelonephritis.

AKI during pregnancy was associated with a higher risk of maternal death. In our study, the mortality rate was 12.7%. The heterogeneity was quite high (*I^2^* = 96.3%), which likely resulted from a higher incidence of sepsis, the main cause of maternal mortality, in developing countries. In contrast, developed countries had a higher incidence of pregnancy-induced hypertension and favorable socioeconomic factors, leading to lower maternal mortality. The mortality rate was approximately 23% to 33%,^[3,29]^ mostly in developing countries, while it was 2.7% to 4.3% in developed countries.^[36]^ Maternal mortality also decreased based on the time period and location of the study. The overall mortality rate declined from 20% in the 1980s to 5.79% in 2013–2014^[37]^

In previous studies, 16% to 73% of pregnant women with AKI required dialysis treatment.^[30]^ In our meta-analysis, approximately 40% of PR-AKI cases received HD, and 10% of cases needed PD. However, the recovery rate of renal function was favorable (70.6%)—only 8.5% of patients were dependent on dialysis after delivery and 14.7% had subsequent chronic kidney disease. Early prevention and treatment should focus more on reducing risk in antenatal care and consider preeclampsia medication in high-risk populations.

The fetal outcome was satisfactory, with survival in 70% cases. There were 28.5% cases of preterm delivery, 18.6% cases of intrauterine death, and 25.4% cases of perinatal infant mortality. Previous studies revealed fetal lost deliveries of 81.3%.^[34]^ The results of the literature reviews in China showed that the incidence of stillbirth or neonatal mortality can reach 30%. The incidence of abortion and stillbirth in this study was nearly equivalent to that reported in previous studies; therefore, improvement in maternal and fetal health is needed.

The merit of the present study is the accumulation of various studies over a long period of time (>30 years), which encompassed >57 million pregnancies. Several meta-analyses have investigated PR-AKI; however, most studies have focused on pregnancy outcomes, such as kidney, maternal, and fetal outcomes. In the present meta-analysis, we attempted to demonstrate the incidence of PR-AKI in a pooled analysis of 40,428 PR-AKI cases. The outcomes in various aspects, especially kidney outcomes, were emphasized. The incidence of PR-AKI in different parts of the world should be considered in the future to help reduce the global burden of AKI.

The following are the limitations of this study. Differences in the definition of AKI due to changes in criteria from different time points and follow-up time contributed to heterogeneous results. In addition, since this study aimed to explore the incidence of PR-AKI, we did not perform subgroup analysis of each variable. Most of the included studies were from the Indian subcontinent, which is associated with a publication bias. Additionally, we were unable to assess long-term kidney-related endpoints.

In conclusion, data obtained from our meta-analysis show that the overall rate of PR-AKI is decreasing worldwide, indicating improvements in and increased awareness of antenatal care. Although the maternal, fetal, and kidney outcomes were favorable, the absolute numbers of deaths and percentage of patients with irreversible kidney damage remained unacceptably high. The most common causes of PR-AKI, including pregnancy-induced hypertension, hemorrhage, and septic abortion/abortion could be prevented, early identified, and treatment. Our analysis serves to encourage future studies to investigate improvements in antenatal care and perform early referral to nephrologists to increase patient safety and mitigate the risk of further kidney injury in PR-AKI.

## Acknowledgments

The authors would like to thank Dr Thanaphan Thitivichienlert for helping with data extraction. We would like to thank Editage (www.editage.com) for English language editing

## Author contributions

Conceptualization: TT, PS

Methodology: PS

Validation: TT, PS

Formal analysis:PS

Resource: TN, TT, PS

Writing manuscript: TT, PS

Project administration: TT

## Supplementary Material



## Footnotes

* World Economic situation and Prospects.
